# ChatGPT and low back pain - Evaluating AI-driven patient education in the context of interventional pain medicine

**DOI:** 10.1016/j.inpm.2025.100636

**Published:** 2025-09-02

**Authors:** Ahmed Basharat, Rohan Shah, Nick Wilcox, Gurpaij Tur, Siddarth Tripati, Prisha Kansal, Niveah Gandhi, Sreekrishna Pokuri, Gabby Chong, Charles A. Odonkor, Narayana Varhabhatla, Robert Chow

**Affiliations:** a Department of Emergency Medicine, Yale Medical Center, USA; b Strategy and Operations, Aetion, USA; c Customer Success, UpSmith AI Solutions, USA; d Mills E Godwin High School Center of Medial Sciences, USA; e Ronald Reagan High School, USA; f Weddington High School, USA; g Memorial High School, USA; h Department of Pain Medicine, Yale Medical Center, USA; i Columbia University, USA; j Yale School of Medicine, USA; k Department of Pain Medicine, University of Colorado Denver, USA

**Keywords:** Low back pain, Artificial intelligence, ChatGPT, Patient questions

## Abstract

**Background:**

ChatGPT and other Large Language Models (LLMs) are not only being more readily integrated into healthcare but are also being utilized more frequently by patients to answer health-related questions. Given the increased utilization for this purpose, it is essential to evaluate and study the consistency and reliability of artificial intelligence (AI) responses. Low back pain (LBP) remains one of the most frequently seen chief complaints in primary care and interventional pain management offices.

**Objective:**

This study assesses the readability, accuracy, and overall utility of ChatGPT's ability to address patients' questions concerning low back pain. Our aim is to use clinician feedback to analyze ChatGPT's responses to these common low back pain related questions, as in the future, AI will undoubtedly play a role in triaging patients prior to seeing a physician.

**Methods:**

To assess AI responses, we generated a standardized list of 25 questions concerning low back pain that were split into five categories including diagnosis, seeking a medical professional, treatment, self-treatment, and physical therapy. We explored the influence of how a prompt is worded on ChatGPT by asking questions from a 4th grader to a college/reference level. One board certified interventional pain specialist, one interventional pain fellow, and one emergency medicine resident reviewed ChatGPT's generated answers to assess accuracy and clinical utility. Readability and comprehensibility were evaluated using the Flesch-Kincaid Grade Level Scale. Statistical analysis was performed to analyze differences in readability scores, word count, and response complexity.

**Results:**

How a question is phrased influences accuracy in statistically significant ways. Over-simplification of queries (e.g. to a 4th grade level) degrades ChatGPT's ability to return clinically complete responses. In contrast, reference and neutral queries preserve accuracy without additional engineering. Regardless of how the question is phrased, ChatGPT's default register trends towards technical language. Readability remains substantially misaligned with health literacy standards. Verbosity correlates with prompt type, but not necessarily accuracy. Word count is an unreliable proxy for informational completeness or clinical correctness in AI outputs and most errors stem from omission, not commission. Importantly, ChatGPT does not frequently generate false claims.

**Conclusion:**

This analysis complicates the assumption that “simpler is better” in prompting LLMs for clinical education. Whereas earlier work in structured conditions suggested that plain-language prompts improved accuracy, our findings indicate that a moderate reading level, not maximal simplicity, yields the most reliable outputs in complex domains like pain. This study further supports that AI LLMs can be integrated into a clinical workflow, possibly through electronic health record (EHR) software.

## 1 Introduction

Pain management remains one of the most challenging aspects of modern healthcare. Over the past two decades, a steady increase in the prevalence and impact of chronic pain has been documented across epidemiologic studies and global burden reports [1]. Among these, low back pain (LBP) has emerged as one of the most common and burdensome conditions in the adult population. In 2012, a comprehensive systematic review published in *Arthritis & Rheumatism* analyzed 165 studies across 54 countries and found that the mean point prevalence of low back pain was approximately 12 %, with a lifetime prevalence nearing 40 % [2]. Despite advances in medications, interventional procedures, and surgical techniques, the global burden of chronic low back pain continues to rise. For example, between 2000 and 2010, prescriptions for both opioid and non-opioid treatments for back and neck pain increased, yet disability-adjusted life years (DALYs) attributed to LBP also grew [3]. Similarly, the number of lumbar spinal fusion surgeries in the U.S. more than doubled from 1998 to 2008 [4]. Importantly, this increase in intervention volume has not consistently led to improved functional outcomes, reinforcing the need for better conservative management and patient education [5].

Given the high prevalence of chronic pain and growing wait times to access pain specialists, physical therapists, and surgeons, empowering patients through accessible education is critical. With the widespread availability of the internet, self-directed health research has become a primary source of information for many individuals managing low back pain. The National Cancer Institute's Health Information National Trends Survey (HINTS) 68.9 % of U.S. adults who sought health information from 2008 to 2017 reported using the internet as their first source during their most recent search [6]. The growing reliance on digital sources has raised concerns regarding the accuracy, readability, and trustworthiness of online medical content. In this context, LLM such as ChatGPT, have emerged as potentially powerful tools for delivering interactive and accessible health education. ChatGPT, a LLM trained on extensive datasets, has demonstrated an ability to produce human-like responses and has been increasingly utilized for patient education across various medical domains.^78^

ChatGPT may provide accurate health information, however the consistency and clinical reliability of its responses, particularly regarding complex conditions like LBP, remains unclear. Moreover, factors such as prompting style, inclusion of references, and readability level may influence the usefulness of chatbot-generated content. Given the widespread public access to ChatGPT and its potential to shape patient understanding, it is necessary to evaluate its utility in the context of low back pain education.

The objective of this study is to evaluate the quality and accuracy of ChatGPT responses to commonly asked questions about low back pain and to determine how prompting strategies influence response correctness, readability, and citation use. As the role of AI in healthcare continues to evolve, such investigations are essential to ensure that digital health tools support informed, safe, and evidence-based patient decision-making.

## 2 Methods

This study was exempt from Institutional Board Review. The study focused on assessing the accuracy, readability, and applicability of responses provided by ChatGPT (Feb 2025, GPT 4.5 version) under different prompting conditions about low back pain.

To assess AI responses, a standardized set of 25 questions was developed and divided into five key categories: Diagnosis (1–5), evaluating the ability to recognize potential pain-related conditions; Medical Professional Guidance (6-10), assessing the appropriateness of recommendations regarding seeking professional care; Treatment (11–15), guidance on pain management strategies, including pharmacologic and non-pharmacologic options; Self-Treatment (16–20), recommendations for over-the-counter (OTC) medications and at-home interventions; and Physical Therapy (21–25), examining responses regarding rehabilitation exercises and long-term pain management strategies. Each question set remained constant across all AI interactions to minimize variability. This research methodology largely mirror work from a paper that utilized AI-generated responses for patient education on thyroid nodules [9].

**Fig. 1:**
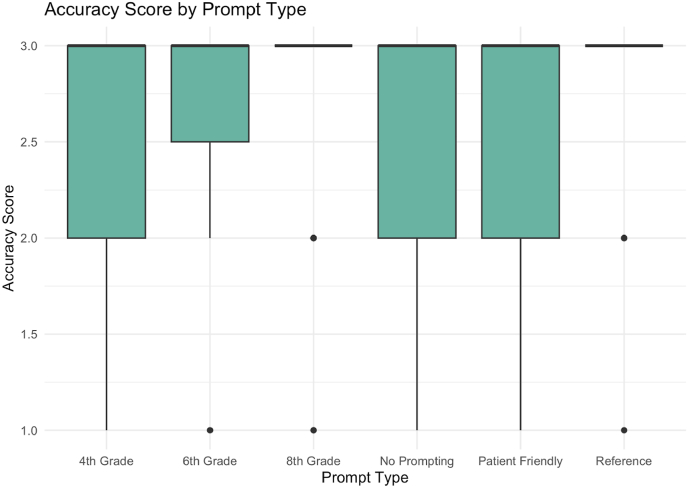
Accuracy score by prompt type.

To explore the influence of prompting on response accuracy and readability ChatGPT was queried under multiple categories. Our first category, No Prompting, consisted of directed patient questions without additional context. The second category, grade specific prompting, assess whether responses varied based on patient demographics, including 4th, 6th, and 8th grade individuals.Table 1, Table 2 lists the prompts and questions utilized for this study. Fig. 1 shows an example of Chat GPT response to questions using 4th grade prompting (see Table 3).

**Table 1:** Types of prompts used.

Prompt Type	Prompt Description
No Prompting	(No special instructions provided)
Patient-Friendly Prompting	I am a patient attempting to learn more about my lower back pain. I am going to ask you twenty-five questions pertaining to my lower back pain. Please use language that would be appropriate for my understanding, but do not compromise on the accuracy of your responses. Be as specific as possible in your answers.
4th-Grade Level Prompting	I am a 4th grader attempting to learn more about my lower back pain. I am going to ask you twenty-five questions pertaining to my lower back pain. Please present your answers at or below the fourth grade United States academic reading level. Do not compromise on the accuracy of your responses. Be as specific as possible in your answers.
6th-Grade Level Prompting	I am a 6th grader attempting to learn more about my lower back pain. I am going to ask you twenty-five questions pertaining to my lower back pain. Please present your answers at or below the sixth grade United States academic reading level. Do not compromise on the accuracy of your responses. Be as specific as possible in your answers.
8th-Grade Level Prompting	I am an 8th grader attempting to learn more about my lower back pain. I am going to ask you twenty-five questions pertaining to my lower back pain. Please present your answers at or below the eighth grade United States academic reading level. Do not compromise on the accuracy of your responses. Be as specific as possible in your answers.
Reference Prompting	I am a patient attempting to learn more about my lower back pain. I am going to ask you twenty-five questions pertaining to my lower back pain. For each answer you provide, make sure that you include statistics or numbers that are relevant. Your answers should come from published medical literature, which you should cite within your answers. Do not compromise on the accuracy of your responses. Be as specific as possible in your answers.

**Table 2:** Questions asked to chat GPT.

**Diagnoses**

1. What are the likely diagnoses of my lower back pain? 2. What would have caused my lower back pain? 3. Could a serious condition be causing my lower back pain? 4. How long will my lower back pain last? 5. What will make my lower back pain worse?

**Medical Professional**

6. Should I see a physician or a medical professional about my lower back pain? 7. Should I visit the emergency room or schedule an appointment for my lower back pain? 8. What type of medical professional should I see for my lower back pain? 9. How often would I need to return to the medical professional about my lower back pain? 10. How soon should I go in to see a medical professional about my lower back pain?

**Treatment**

11. Are pain medications appropriate for this lower back pain? 12. What are treatment options that a medical professional would recommend? 13. What are the potential side-effects to pain medications for lower back pain? 14. Are there other professional options for treatment besides a medical professional? 15. How long does treatment for lower back pain usually last?

**Self-Treatment**

16. What are things I can do to prevent lower back pain in the future? 17. What over-the-counter (OTC) drugs are appropriate for me to take for lower back pain? 18. How long should I take these OTC drugs for lower back pain? 19. Do you recommend special accommodations in lifestyle for my lower back pain? 20. What are at-home therapies I can use for my lower back pain?

**Physical Therapy**

21. Will I need to regularly see a physical therapist after treatment? 22. How often would I need to see a physical therapist for my lower back pain? 23. Will physical exercises on my own remedy this lower back pain? 24. What are physical exercises I can do on my own to treat my lower back pain? 25. How often should I conduct these physical exercises for my lower back pain?

**Table 3:** Example of two questions and responses from 4th grade prompt.

**Question 1:**	What are the likely diagnoses of my lower back pain?
**Response 1:**	You might have a muscle strain, poor posture, or a small joint problem. These are common reasons kids and adults get lower back pain.
**Question 2:**	What would have caused my lower back pain?
**Response 2:**	You may have lifted something heavy, played too hard, sat too long, or slept in a weird position. These things can hurt the muscles in your back.

Responses were evaluated for medical accuracy by an independent panel of medical professionals, including an attending, a fellow, and resident physician. AI-generated responses were graded using a hierarchical three-tier scale: Score 1 (Incorrect), responses contained misinformation or were inconsistent with established medical guidelines; score 2 (Partially Correct), response was vague, incomplete, or lacked critical details; score 3 (Correct), response was accurate. Each answer was graded by using International Pain and Spine Intervention Society (IPSIS) guidelines. The three reviewers were blinded to each other's ratings to prevent bias in the evaluation process. Each reviewer scored independently without prior discussion. If discrepancies in the ratings occurred, the attending physician made the final decision to resolve the differences.

Readability and comprehensibility of responses were assessed using established text evaluation metrics. The Flesch-Kincaid Grade Level was calculated to estimate the U.S. academic reading level required to understand each response. A chi-squared analysis was used to compare the proportion of correct and incorrect across different prompt conditions. The percentage of correct responses is determined by counting how many responses were rated as “Correct” by the reviewers. This total is then divided by the overall number of responses to compute the proportion of accurate responses.

Additionally, a one-way analysis of variance (ANOVA) was performed to analyze differences in readability scores, word count, and response complexity across prompting conditions. Correlations between readability scores and medical accuracy were also examined to assess whether responses written at lower reading levels contained more or fewer inaccuracies. The study findings will be presented using data visualization techniques, including tables and graphical representations, to highlight trends in AI-generated responses. Specifically, the analysis will explore whether different patient literacy levels (as simulated through age-specific prompting) receive different levels of accuracy or readability in AI-generated medical information. The prompt did not simulate other demographic factors such as language proficiency, socioeconomic status, or culture background. Furthermore, insights from this study will be contextualized within the broader discourse on AI's role in patient education and clinical decision-making. This study does not involve direct human subjects, as it solely analyzes AI-generated responses. However, given the increasing use of AI as a supplementary tool for patient education, the findings will be interpreted with an emphasis on responsible AI implementation in medical settings. The study aims to inform clinicians about the reliability of ChatGPT for patient education in pain treatment and to provide recommendations on its appropriate use in healthcare communication.

## 3 Results

### 3.1 Objective statistics

1. Prompt Type Influences Accuracy in Statistically Significant Ways

A one-way ANOVA, prompt type was significantly associated with accuracy score (F(5, 444) = 2.45, *p* = 0.033). While the overall mean accuracy across all prompts was high (mean = 2.81 out of 3), the variance in correctness between prompt types was non-trivial. In Fig. 1, the bars represent the mean accuracy score for each prompt type, and the vertical lines denote the 95 % confidence intervals. Both the 8th Grade and Reference prompt groups have near-ceiling accuracy scores (3.0), resulting in very small CIs that may be visually minimal in the plot.

The 4th Grade prompts produced the most variable responses, with scores ranging from 1 (incorrect) to 3 (fully correct). The interquartile range (IQR) in Fig. 1 spans the full spectrum of the accuracy scale, with multiple low outliers. The 6th Grade prompts reduced variance, though still included a small number of score-1 responses. In contrast, the Reference, No Prompting, and Patient-Friendly formats exhibited tight distributions clustered at the ceiling (score = 3), with negligible incidence of inaccurate outputs. Reference prompts achieved a median and mode of 3, with only one score of 2 and one of 1 across all observations.

Tukey's HSD test confirmed that 4th Grade prompts were significantly different from Reference and No Prompting conditions, with estimated mean differences of −0.32 (95 % CI: 0.57 to −0.06, *p* = 0.011) and −0.29 (95 % CI: 0.54 to −0.04, *p* = 0.018), respectively. These findings suggest that over-simplification of ChatGPT's responses (e.g., to a 4th-grade level) degrades ChatGPT's ability to return clinically complete responses. In contrast, Reference and neutral queries preserve accuracy without additional engineering.

2. Readability Remains Substantially Misaligned with Health Literacy Standards

Across all prompt types, readability (measured by Flesch–Kincaid grade level) often exceeded recommended thresholds for patient-directed materials (≤6th grade). Reference prompts had a median grade level of 14.3 (IQR: 12.5–17.1), indicating language appropriate for undergraduate education or higher. Patient-Friendly prompts, though engineered for accessibility, still yielded a median grade level of 13.2. Only 4th Grade prompts consistently generated responses below the 8th grade level (median: 5.8), though this came at the cost of accuracy.

Despite explicitly prompting ChatGPT to generate responses at a 6th or 8th grade reading level, the Flesch–Kincaid analysis showed that outputs consistently exceeded the targeted complexity: 6th grade prompt group: mean FK grade level = 8.30 (SD = 4.47); 8th grade prompt group: mean FK grade level = 8.06 (SD = 3.95). Only the 4th grade prompt conditions consistently produced outputs below the 8th grade threshold. This pattern suggests a limitation in the model's ability to self-regulate reading level based solely on prompt instructions, a finding consistent with prior research on AI-generated patient education materials.

Statistical testing via one-way ANOVA showed significant differences in FK scores across prompt types (F(5, 444) = 11.62, *p* < 0.001), with post-hoc contrasts confirming Reference and Patient-Friendly responses were significantly more complex than 4th–8th grade formats.Despite prompt-level simplification, ChatGPT's default register trends toward technical language. The model lacks intrinsic awareness of health literacy boundaries unless explicitly constrained, and even then, its outputs often overshoot acceptable reading levels.

3. Verbosity Correlates with Prompt Type, But Not Necessarily With Accuracy

As shown in Fig. 4, Reference prompts yielded the longest responses (median = 35 words, max = 97), significantly exceeding all other conditions (*p* < 0.001) (see Fig. 3). However, this verbosity did not confer meaningful gains in accuracy, suggesting diminishing returns.

For example, the 6th Grade prompts, with a median word count of just 19 words, achieved an average accuracy of 2.84. In comparison, Reference prompts, with nearly double the length, only marginally exceeded that (mean accuracy = 2.87). Word count is an unreliable proxy for informational completeness or clinical correctness in AI outputs. This reinforces the need for structured content evaluation beyond surface-level metrics.

4. Most Errors Stem from Omission, Not Commission

**Fig. 2:**
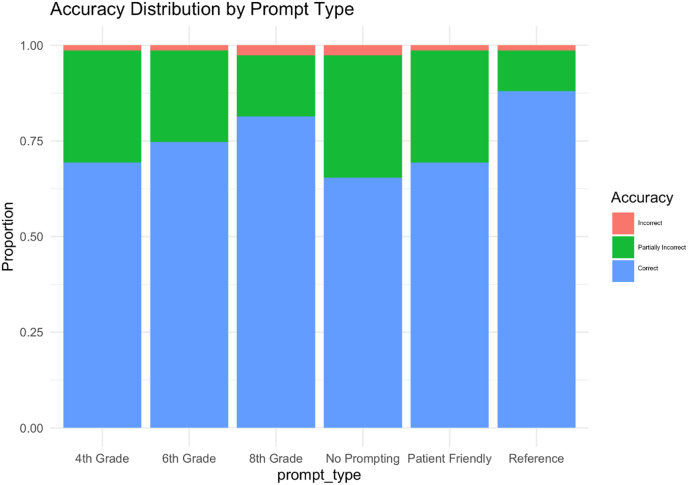
Accuracy distribution by prompt type.

Fig. 2 illustrates the proportion of outputs across each accuracy tier. Across all prompt types, less than 6 % of responses were rated as fully incorrect (score = 1). A substantial portion (18–30 %, depending on prompt) were rated as partially correct (score = 2), typically due to omission of key safety considerations or incomplete diagnostic explanations. Reference and 8th Grade prompts produced the fewest incomplete responses (12 % and 14 %, respectively). ChatGPT does not frequently hallucinate false claims in pain medicine — instead, it more often fails to meet the clinical threshold for completeness. This “sins of omission” pattern mirrors human medical errors and poses distinct safety risks.

5. Emerging Narrative

**Fig. 3:**
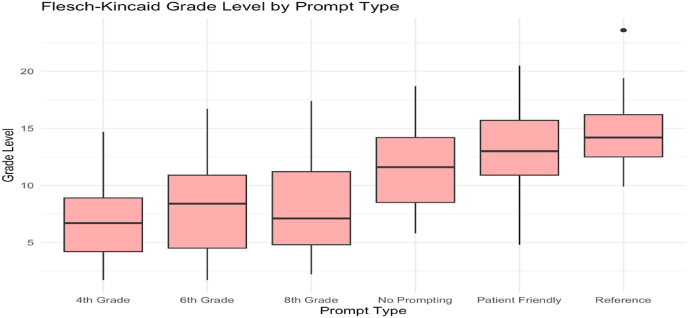
Flesch-Kincaid grade level by prompt type.

**Fig. 4:**
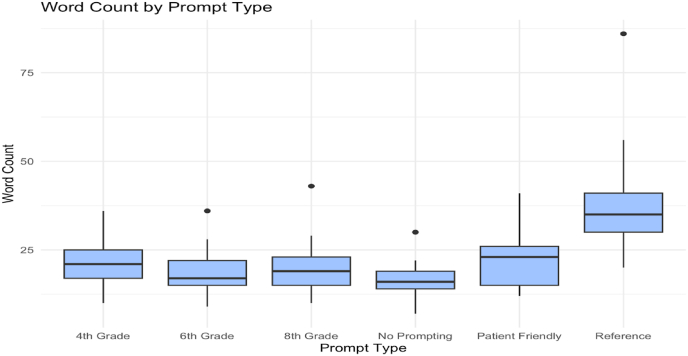
Word count by prompt type.

This analysis complicates the assumption that “simpler is better” in prompting LLMs for clinical education. Whereas earlier work in structured conditions (e.g., thyroid nodules) suggested that plain-language prompts improved accuracy, our findings indicate that a moderate reading level — not maximal simplicity — yields the most reliable output in complex domains like pain.

Moreover, the minimal performance difference between Reference and “No Prompting” formats implies that ChatGPT defaults to reasonable clinical scaffolds when left unprompted but can be derailed by prompts that are too simplified or too ambiguous.

## 4 Discussion

This study evaluated AI-generated responses to common questions about LBP across different prompt types. The authors chose ChatGPT for this study because of its widespread use, rapid growing presence and its increase in practice in a clinical setting. Overall, ChatGPT performed high with an average accuracy score of 2.81/3 with less than 6 % of responses rated as fully incorrect. This is consistent with earlier studies in thyroid nodules and sleep apnea, which have shown high accuracy rates with appropriate prompting [7,8]. ChatGPT accuracy relies heavily on prompt type. A one-way ANOVA revealed that prompt type played a role in how accurate responses could be (F(5, 444) = 2.45, p = 0.033). This raises an important point, as the general public may lack knowledge of AI producing different answers based on how the question is asked.

While prompt type influenced response accuracy, it also significantly impacted readability (F(5, 444) = 11.62, *p* < 0.001). The readability of responses from GPT 4.0 mostly misaligned with health literacy guidelines. The National Institutes of Health recommend that patient education materials be written at an eighth-grade level [10]. In our analysis, only prompts stated for a fourth grade reading level produced responses that are within these health literacy guidelines (median FK grade level of 5.8), however, this prompting reduced the accuracy of the responses. The rest of the prompts had outputs at the collegiate reading levels, which is significantly higher than NIH recommendations. This finding is similar to prior studies that show AI and medical websites have frequently fail to meet literacy standards [11,12].

Verbosity was affected by prompt type. Reference prompts had the longest answers, with a median of 35 words and maxing nearly 100 words, significantly exceeding all other prompt styles (p < 0.001). Longer answers were not necessarily more accurate, even when compared to shorter prompts designed for a 6th-grade level. 6th-grade level prompts average about 19 words and were relatively as accurate as reference prompts. These results may suggest that longer answers do not guarantee better information. As mentioned before, some earlier studies may have suggested that plain-language prompts could improve accuracy [7,8]. Our finding suggests that using moderate reading level, such as 8th grade level, produces more reliable responses, consistent with National Institute of Health recommendations [10]. Moving forward, it will be important to find ways to keep responses clear and useful, without unnecessary length that could otherwise confuse patients.

ChatGPTs mistakes were mostly from missing information, not false statements. Between 18 % and 30 % of responses were partially correct because the chatbot left out key safety points or didn't fully explain answers. This can be comparable to how current providers sometimes forget to mention certain information. Only less than 6 % of responses were actually completely incorrect. It's reassuring that GPT 4.0 didn't often produce blatantly wrong answers, however incomplete responses could still be misleading to patients without guidance from a medical professional [13].

## 5 Limitations

This study has several limitations. While the question set was derived from common patient concerns, it is not exhaustive and may not reflect the full range of LBP presentations. We did not evaluate patient comprehension or behavioral outcomes in response to ChatGPT's advice metrics. Our analysis was confined to GPT-4.0. Future versions (e.g., GPT-4.5, GPT-5, Claude, Bard) may yield different results. Additionally, prompt structure was predefined rather than dynamically tailored.

## 6 Conclusion

In summary, this study highlights the importance of prompt design when using ChatGPT to answer clinical questions about low back pain. While the model can produce mostly accurate responses, simplification efforts, especially when extreme, may reduce accuracy and omit important safety information. ChatGPT should be used cautiously as a supplemental resource rather than a standalone tool for patient education in pain contexts. Future development of adaptive prompting strategies and real-time readability controls may improve safety and usability in diverse populations.

## Contribution breakdown by category

Idea Generation: Robert Chow, Narayana Varhabhatla, Charles Odonkor

Manuscript Drafting: Ahmed Basharat, Sreekrishna Pokuri, Robert Chow.

Manuscript Editing and Revisions: Ahmed Basharat, Sreekrishna Pokuri, Robert Chow.

Outline and Prompting: Nick Wilcox (lead), Gabby Chong (assisted).

Methodology Design: Robert Chow, Nick Wilcox.

Data Collection: Ahmed Basharat, Sreekrishna Pokuri, Robert Chow.

Statistical Analysis and Results: Rohan Shah, Sreekrishna Pokuri.

Discussion and Conclusion Assistance: Gurpaij Tur, Siddharth Tripathi, Prisha Kansal, Niveah Gandhi.

## Declarations of interests

All authors have no conflict of interests.

## References

[1] Vasudevan S. (2015).

[2] Hoy D., March L., Brooks P. (2014). The global burden of low back pain: estimates from the global burden of disease 2010 study. Ann Rheum Dis.

[3] Wu A., March L., Zheng X. (2020). Global low back pain prevalence and years lived with disability from 1990 to 2017: estimates from the global burden of disease study 2017. Ann Transl Med.

[4] Rajaee S.S., Bae H.W., Kanim L.E.A., Delamarter R.B. (2012). Spinal fusion in the United States: analysis of trends from 1998 to 2008. Spine.

[5] Mafi J.N., McCarthy E.P., Davis R.B., Landon B.E. (2013). Worsening trends in the management and treatment of back pain. JAMA Intern Med.

[6] Finney Rutten L.J., Blake K.D., Greenberg-Worisek A.J., Allen S.V., Moser R.P., Hesse B.W. (2019). Online health information seeking among US adults: measuring progress toward a healthy people 2020 objective. Publ Health Rep.

[7] Ayers J.W., Poliak A., Dredze M. (2023). Comparing physician and artificial intelligence chatbot responses to patient questions posted to a public social media forum. JAMA Intern Med.

[8] Sarraju A., Bruemmer D., Van Iterson E., Cho L., Rodriguez F., Laffin L. (2023). Appropriateness of cardiovascular disease prevention recommendations obtained from a popular online chat-based artificial intelligence model. JAMA.

[9] Campbell D.J., Estephan L.E., Sina E.M. (2024). Evaluating ChatGPT responses on thyroid nodules for patient education. Thyroid.

[10] Rooney M.K., Santiago G., Perni S. (2021). Readability of patient education materials from high-impact medical journals: a 20-Year analysis. J Patient Exp.

[11] Cimbek E.A., Cimbek A. (April 2023). Online health information on thyroid nodules: do patients understand them?. Minerva Endocrinol.

[12] Jindal P., MacDermid J. (2017). Assessing reading levels of health information: uses and limitations of flesch formula. Educ Health.

[13] Athaluri SA, Manthena SV, Kesapragada VSRKM, Yarlagadda V, Dave T, Duddumpudi RTS. Exploring the boundaries of reality: investigating the phenomenon of artificial intelligence hallucination in scientific writing through ChatGPT references. Cureus. Published online April 11, 2023. doi:10.7759/cureus.37432. 10.7759/cureus.37432 PMC10173677 37182055

